# Mice with altered BDNF signaling as models for mood disorders and antidepressant effects

**DOI:** 10.3389/fnbeh.2014.00143

**Published:** 2014-04-30

**Authors:** Jesse S. O. Lindholm, Eero Castrén

**Affiliations:** Neuroscience Center, University of HelsinkiHelsinki, Finland

**Keywords:** Bdnf deficient mice, transgenic, TrkB signaling, behavior, depression, neurotrophins, BDNF

## Abstract

Brain-derived neurotrophic factor (BDNF) and its receptor tyrosine kinase TrkB support neuronal survival during development and promote connectivity and plasticity in the adult brain. Decreased BDNF signaling is associated with the pathophysiology of depression and the mechanisms underlying the actions of antidepressant drugs (AD). Several transgenic mouse models with decreases or increases in the amount of BDNF or the activity of TrkB signaling have been created. This review summarizes the studies where various mouse models with increased or decreased BDNF levels or TrkB signaling were used to evaluate the role of BDNF signaling in depression-like behavior. Although a large number of models have been employed and several studies have been published, no clear-cut connections between BDNF levels or signaling and depression-like behavior in mice have emerged. However, it is clear that BDNF plays a critical role in the mechanisms underlying the actions of AD.

The neurotrophin brain-derived neurotrophic factor (BDNF) supports neuronal survival during development and promotes connectivity and plasticity in the adult brain by activating its tyrosine kinase receptor TrkB (Park and Poo, 2013). Impaired BDNF signaling through TrkB is associated with the pathophysiology of mood disorders and neuronal plasticity promoted by BDNF underlies some of the actions of antidepressant drugs (AD) (Duman and Monteggia, 2006; Castrén et al., 2007). BDNF mRNA and protein levels in the rodent hippocampus (HC) correlate with depressive behaviors and with anatomical changes in the HC induced by stress (Duman et al., 1997; Duman and Monteggia, 2006). Similar findings have also been reported in the brains of depressed patients (Sheline et al., 1996; Bremner et al., 2000).

Direct injection of BDNF into the dentate gyrus (DG) or CA3 regions of the HC, which are brain regions implicated in spatial memory and in the regulation of emotionality and mood, leads to antidepressant-like effects and to enhancement of the antidepressant-like activity of paroxetine in rodent behavioral despair models (Shirayama et al., 2002; Deltheil et al., 2008). Tyrosine kinase inhibitor K252a blocks this effect, suggesting that the antidepressant-like behavior of BDNF is dependent on TrkB activity (Shirayama et al., 2002). Similar antidepressant-like behavioral effects were observed when BDNF was injected into the midbrain region, which contains serotonergic neurons projecting to forebrain regions (Siuciak et al., 1997). Furthermore, peripheral administration of BDNF produces an antidepressant-like effect similar to the effects observed with intracranial administration (Schmidt and Duman, 2010). By contrast, direct injection of BDNF into the ventral tegmental area, a brain area that contains dopaminergic cell bodies, causes depression-like behavior, and blockade of BDNF signaling in the nucleus accumbens, a brain area involved in reward and hedonic behavior, produces antidepressant-like behavior (Eisch et al., 2003; Berton et al., 2006). Therefore, the effects of BDNF on depression-like behavior depend upon the functional role of the circuitry that is targeted.

Chronic treatment with ADs, electroconvulsive shock treatment, and physical exercise all increase BNDF mRNA and protein levels in both animals and humans (Nibuya et al., 1995; Zetterström et al., 1998; Russo-Neustadt et al., 1999; Chen et al., 2001; Coppell et al., 2003; Duman et al., 2008; Marais et al., 2009). Importantly, chronic treatment with ADs restores stress-induced changes in BDNF mRNA levels in experimental animals (Nibuya et al., 1995; Duman and Monteggia, 2006). However, there is evidence that the regulation of BDNF expression by ADs is more complex. Some studies report that BDNF mRNA levels decrease shortly after AD administration (Coppell et al., 2003; Kozisek et al., 2008). Furthermore, different classes of ADs activate TrkB signaling after both acute and long-term administration (Saarelainen et al., 2003; Rantamäki et al., 2007), and this TrkB mediated BDNF signaling is essential for the antidepressant-like effects of ADs in rodents (Saarelainen et al., 2003; Ibarguen-Vargas et al., 2008).

## BDNF and human mood disorders

A comprehensive review of the role of BDNF in mood disorders is beyond the scope of this review, but some remarks are useful to provide a perspective for the rodent data reviewed below. BDNF and TrkB receptor levels in the brains and BDNF concentrations in the plasma of suicidal subjects are decreased (Dwivedi et al., 2003; Kim et al., 2007). Furthermore, AD treatment increases BDNF levels in the HC of postmortem human brains (Chen et al., 2001).

A single nucleotide polymorphism (SNP) in the human BDNF gene in which valine (Val) is substituted with methionine (Met) in codon 66 (Val66Met) reduces activity-dependent BDNF release and impairs hippocampal function (Egan et al., 2003). This SNP is only observed in humans and is expressed throughout the general population (Val/Met: 20–50%, Met/Met 3–20%), but is more common in Asian than in Caucasian populations (Verhagen et al., 2008). Heterozygous Met allele carriers have smaller hippocampal volumes and perform worse in hippocampal-dependent memory tasks than people with the Val/Met allele (Egan et al., 2003). No clear connections exist between this SNP and clinical depression or anxiety (Gratacos et al., 2007; Verhagen et al., 2008; Gyekis et al., 2013). However, there is evidence for a higher sensitivity to stressful life events among Met-allele carriers (Kaufman et al., 2006; Hosang et al., 2014). Unexpectedly, heterozygous met-allele carriers show a better response to antidepressant drug treatments than homozygous (Val/Val or Met/Met) carriers (Niitsu et al., 2013). By contrast, a mouse model of the Val66Met polymorphism suggests an impaired response to antidepressants in Met carrying mice (Chen et al., 2006). Potential associations between the Val66Met SNP and other mental disorders, such as substance abuse, eating disorders and schizophrenia have, however, been reported (Gratacos et al., 2007; Eisenberg et al., 2013).

BDNF is abundantly expressed in blood platelets, therefore, BDNF levels in human serum are relatively high (Karege et al., 2005). Several clinical studies demonstrate that BDNF serum levels are decreased in depressed patients and that these levels recover following successful antidepressant treatment (Karege et al., 2005; Monteleone et al., 2008; Sen et al., 2008). Unfortunately, mouse blood platelets appear to contain little or no BDNF and, consequently, BDNF levels in mouse serum are low. This species difference has greatly hampered mechanistic studies into this interesting and consistent finding of the relationship between mood disorders and serum BDNF levels.

## Genetic mouse models of altered BDNF and TrkB

A substantial number of genetically modified mouse models with altered BDNF expression or TrkB signaling have been produced. There are several distinct approaches that have been taken to genetically modify BDNF signaling. First, the most common method involves knock down of BDNF expression from either the whole organism or from a spatially restricted region. Second, TrkB signaling is inhibited through either TrkB knockout (KO) or other strategies that decrease TrkB activity. Third, there are gain-of-function models that enhance either BDNF expression or TrkB activity. This review will summarize the results from these BDNF and TrkB genetic models that are related to mood disorders.

### Decreased BDNF levels

Ernfors et al. (1994) produced mutant mice lacking BDNF, the constitutive BDNF KO, in the early 1990s. Homozygous BDNF KO mice have severe health problems and die soon after birth (Ernfors et al., 1994). However, heterozygous BDNF KO [BDNF^(±)^] mice are vital and fertile. Their BDNF mRNA and protein levels in brain are approximately 50% of their wildtype littermates (Korte et al., 1995; MacQueen et al., 2001; Ibarguen-Vargas et al., 2009). This deficiency strongly down regulates the early postnatal signaling capacity of TrkB receptors. However, in adult mice, TrkB phosphorylation appears to be only slightly altered, if at all (Rantamäki et al., 2011). Similarly, forebrain-specific conditional BDNF KO mice have normal TrkB phosphorylation levels (Rantamäki et al., 2011). During normal postnatal development, the responsiveness of TrkB to BDNF is decreased, and TrkB simultaneously gains the ability to be activated by BDNF-independent transactivating mechanisms (Knüsel et al., 1997; Di Lieto et al., 2012). The reason and significance of these findings are currently unknown but it is possible that BDNF-independent TrkB activation mechanisms, such as zinc or adenosine induced transactivation (Lee and Chao, 2001; Huang et al., 2008), predominate in the adult brain.

The behavioral phenotypes of BDNF knock down mice are widely studied; however, results vary from one laboratory to another (Table 1). BDNF^(±)^ mice have increased appetite and are usually obese (Lyons et al., 1999; Kernie et al., 2000; Chen et al., 2006). These mice appear to have normal visual, auditory, and nociceptive senses (Liu et al., 2004; Bath et al., 2008); however, they have olfactory system impairments (Liu et al., 2004; Bath et al., 2008). When tested in a novel environment, the locomotor activity of these mice does not differ from their wildtype littermates (Lyons et al., 1999; MacQueen et al., 2001; Chourbaji et al., 2004; Chen et al., 2006; Ibarguen-Vargas et al., 2009; Marais et al., 2009; Lindholm et al., 2012; Psotta et al., 2013; however see Kernie et al., 2000). BDNF^(±)^ mice also show mild impairments in learning and memory. They display impaired contextual memory but have normal cued memory (Linnarsson et al., 1997; Liu et al., 2004; Chen et al., 2006). BDNF^(±)^ mice also display impaired fear extinction behavior in a fear conditioning paradigm (Psotta et al., 2013). However, these findings are not consistent throughout all studies (Chourbaji et al., 2004; Uutela et al., 2012). Importantly, there are humans with heterozygous deletions of BDNF or TrkB, and these people are obese and suffer from mental retardation (Yeo et al., 2004; Gray et al., 2006).

**Table 1:** **Behavioral results of heterozygous BDNF-KO mice**.

**Behavioral tests**	**Result**	**Background**	**Reference**
BW, LA and FI	Increased appetite and weight, and decreased locomotor activity in BDNF^(±)^ mice, but not in heterozygous NT4/5, NT3, TrkC, or TrkA knockout mice	C57Bl/6 and 129Sv mixed F2 background	Kernie et al., 2000
Anhedonia, EPM, FST, LH, NOR, OF, PA, SP, staircase	No differences in most of tests. Longer escape latencies in LH	C57Bl/6 and 129Sv mixed background	MacQueen et al., 2001
NSF, OF, RI, TST	No behavioral changes, effects of ADs blocked in heterozygous mice	C57Bl/6 and 129Sv mixed background	Ibarguen-Vargas et al., 2009
EZM, FC, FST, LD, novel cage, OF, RR	No behavioral changes	C57Bl/6 and 129Sv mixed background	Chourbaji et al., 2004
EPM, OF	Increased sensitivity to pre/postnatal maternal environment (high or low maternal care)	C57Bl/6J and/or BALB/c	Carola and Gross, 2010
BW, FI, OF, RI	Increased appetite, weight and aggressiveness	C57Bl/6	Lyons et al., 1999
BW, EPM, FC, LA, NSF, OF, RI	Increased weight, aggressiveness, and anxiety-like behavior, impaired contextual but intact cue memory. No changes in locomotor activity	C57Bl/6J	Chen et al., 2006
FST	No behavioral changes, effects of ADs blocked in heterozygous mice	129Sv X BALB/c	Saarelainen et al., 2003
FC, pain, vision and auditory tests	No changes in senses or baseline freezing, impairment in contextual memory-, but not in cue memory test	C57Bl/6	Liu et al., 2004
FC, OF	No changes in locomotor activity, but adult animals have impaired fear extinction learning	C57Bl/6J	Psotta et al., 2013
Spontaneus olfactory discrimination	Impairment in olfactory system	C57Bl/6	Bath et al., 2008
FST	Depression-like behavior after stress or MEK inhibitor	C57Bl/6?	Duman et al., 2007
EPM, OF	Increased anxiety-like behavior, no changes in locomotor activity	C57Bl/6	Li et al., 2010
ASR, BW, FC, OF, RR, WM	No behavioral changes, increased weight	C57Bl/6	Uutela et al., 2012
WM	Impaired learning and memory	129/J x BALB/c	Linnarsson et al., 1997
FST, OF	No behavioral changes, effects of ADs blocked in heterozygous mice	C57Bl/6J	Lindholm et al., 2012

Abbreviations: ASR, Acustic startle response; BW, Body weight; EPM, elevated plus-maze; FC, fear conditioning; FI, food intake; FST, forced swimming test; EZM, elevated zero maze; LA, locomotor activity; LD, light-dark box; LH, learned helplessness; NOR, novel object recognition; NSF, novelty suppressed feeding; OF, open field; PA, passive avoidance; RI, resident-intruder test; RR, RotaRod; SP, sucrose preference; TST, tail suspension test; WM, water maze.

The anxiety- and depression-like behavioral phenotypes of these mice are even more complex. Some of the studies found that anxiety-like behaviors of BDNF^(±)^ mice is indistinguishable from littermate controls (Chourbaji et al., 2004; Ibarguen-Vargas et al., 2009; Lindholm et al., 2012), whereas others found that these mice are more aggressive and display increased anxiety (Lyons et al., 1999; Chen et al., 2006; Li et al., 2010). These mice do not show depression-like behavior in models of behavior despair (such as the forced swim or tail suspension tests) (MacQueen et al., 2001; Saarelainen et al., 2003; Chourbaji et al., 2004; Duman et al., 2007; Ibarguen-Vargas et al., 2009), but they do show depression-like behavior in the learned helplessness test (MacQueen et al., 2001). Furthermore, when exposed to stress, BDNF^(±)^ mice demonstrate an anxiety- and depression-like behavioral phenotype (Duman et al., 2007; Carola and Gross, 2010). Several studies have consistently shown that BDNF^(±)^ mice are resistant to classical ADs in models of behavioral despair (Saarelainen et al., 2003; Ibarguen-Vargas et al., 2009; Lindholm et al., 2012). Thus, BDNF^(±)^ mice do not show any clear baseline anxiety- or depression-like behaviors, but they are more vulnerable to stress and the effects of ADs are blocked in these mice.

Spatially restricted BDNF KO mouse lines have been produced by crossing mice carrying floxed BDNF (flBDNF) alleles with lines expressing Cre recombinase under a tissue-specific promoter (Table 2). Mood-related behaviors have been investigated using Cre lines directed by the α-calcium/calmodulin-dependent protein kinase II (CamK) promoter, which drives expression in postmitotic neurons (Rios et al., 2001). These mice showed increased food intake, weight gain, anxiety-like behavior, and hyperactivity (Rios et al., 2001). Monteggia et al. (2007) also investigated two conditional BNDF KO mice lines. One line crossed flBDNF mice with human glial fibrillary acidic protein (GFAP)-Cre mice and the other with CaMKII-Cre. In GFAP-Cre mice, Cre recombinase is broadly expressed in forebrain regions during late embryogenesis, and in CamK-Cre mice it is expressed in similar regions during postnatal development (Monteggia et al., 2007). BDNF mRNA levels in the CamK-Cre x flBDNF mice were significantly reduced in the HC and the dorsal cerebral cortex, while GFAP-Cre x flBDNF mice lacked BDNF mRNA in a nearly identical pattern. Male conditional KOs from both lines showed hyperactivity but normal depression-like behavior. Female mice displayed normal activity scores but had increased depression-like behavior. Furthermore, ADs failed to have any effect in either sex. Chan et al. (2006) also produced two conditional BDNF KO mice lines, crossing flBDNF mice with either Nestin or CamK-Cre, which resulted in pre- and postnatal recombination, respectively. The authors found that both BDNF KO mouse lines displayed hyperactivity and aggressive behavior. In addition, both BDNF KO mouse lines displayed depression-like behavior in the tail suspension test, but lower immobility scores in the forced swim test when compared to wildtype littermates.

**Table 2:** **Behavioral results of transgenic BDNF mice**.

**Mutant mice**	**Behavioral tests**	**Result**	**Background**	**Reference**
Inducible BDNF-KO in forebrain: adult KO	BW, FC, LA, RI, pain test	No changes in BW, LA, aggressiviness, cue memory or pain sensitivity, impaired contextual memory	Mixed background: BL6/SJL × ICR × ICR × Bl6/sv129	Monteggia et al., 2004
Inducible BDNF-KO in forebrain: early KO	BW, FC, FST, LA, RI	No changes in BW or aggressiviness, hyperactive phenotype, impaired contextual and cue memory	Mixed background: BL6/SJL × ICR × ICR × Bl6/sv129	Monteggia et al., 2004
Inducible BDNF-KO in forebrain	BW, FST, LA, NSF, OF, SP, TST	BDNF-KO increases vulnerability to chronic stress induced anxiogenic and anhedonic behaviors in female, but not in male mice	Mixed background: BL6/SJL × ICR × ICR × Bl6/sv129	Autry et al., 2009
Inducible BDNF-KO in forebrain	FST	Antidepressant-like effect of ketamine was blocked	Mixed background: BL6/SJL × ICR × ICR × Bl6/sv129	Autry et al., 2011
Conditional BDNF-KO: postnatal brain	BW, FI, LA, LD	Increased appetite, weight and anxiety-like behavior, hyperactivity	Mixed background	Rios et al., 2001
Conditional BDNF-KO	LA, EPM, FST, OF, SP	Hyperactivity in male mice, depression-like behavior in female mice. Effects of ADs blocked in both sex in FST	–	Monteggia et al., 2007
BDNF (Val66Met) polymorphism	EPM, FC, LA, NSF, OF, RI	Increased weight, aggressiveness, and anxiety-like behavior, impaired contextual but intact cue memory. No changes in locomotor activity	C57Bl/6J	Chen et al., 2006
BDNF (Val66Met) polymorphism	Spontaneus olfactory discrimination	Impairment in olfactory system	C57Bl/6	Bath et al., 2008
BDNF (Val66Met) polymorphism	EPM, FST, NSF, OF, SP, TM, WM	Depression- and anxiety-like behavior, poor spontaneus alteration only after stress. Rescuing effect of desipramine but not fluoxetine in FST	–	Yu et al., 2012
BDNF (Val66Met) polymorphism	EPM, OF	Increased anxiety-like behavior, no changes in locomotor activity. Music rescues anxiogenic behavior	C57Bl/6	Li et al., 2010
BDNF overexpressing	WM	Heterozygous but not homozygous mice showed improved learning and memory	C57Bl/6J	Nakajo et al., 2008
BDNF overexpressing in exitatory neurons in forebrain	EPM, FST, OF	Anxiogenic- and antidepressant-like behavior. No changes in locomotor activity	C57Bl/6	Govindarajan et al., 2006
BDNF overexpressing, hemizygous	ASR, EPM, FC, FST, LD, OF, PPI, RR, SA, TM, TST	Impaired working memory, but normal contextual and cue memory, impairments in ASR and PPI, anxiety-like behavior in LD but not in EPM. Normal motor and locomotor function, no changes in depression-like behavior	C57Bl/6J	Papaleo et al., 2011

Abbreviations: ASR, Acustic startle response; BW, body weight; EPM, elevated plus-maze; FC, fear conditioning; FI, food intake; FST, forced swimming test; LA, locomotor activity; LD, light-dark box; NSF, novelty suppressed feeding; OF, open field; PPI, pre-pulse inhibition; RI, resident-intruder test; RR, rotarod; SA, social approach; SP, sucrose preference; TST, tail suspension test; TM, T-maze; WM, water maze.

Inducible mice with temporally restricted manipulations of BDNF have also been made. In these mice, a pharmacological compound, such as tamoxifen or doxycycline, is used to either activate or inactivate gene expression. Monteggia et al. (2004) used this method to produce triple transgenic (NSE-tTA × TetOp-Cre × floxed BDNF) mice in which BDNF expression can be knocked out upon withdrawal of doxycycline from the diet. When doxycycline was absent during early development, these mice showed a hyperactive phenotype and impairments in contextual and cued memory tasks. By contrast, when doxycycline was absent during adulthood these mice had a milder phenotype only showing some impairments in contextual memory tasks. In addition, mice with adulthood BDNF knock down do not show any baseline depression-like behaviors in behavioral despair models, however, antidepressant-like effects are lost (Monteggia et al., 2004; Autry et al., 2011). Autry et al. (2009) also examined the effects of chronic unpredictable stress (CUS) on similar mice. The authors found that female mice with induced BDNF knock down were more vulnerable to CUS-induced anxiogenic and anhedonic behaviors while male knock down mice did not differ from wildtype littermates.

To study more localized effects of BDNF deletion, Adachi et al. (2008) injected adeno-associated virus (AAV)-driven Cre into either the DG or CA1 area of flBDNF mice to specifically delete BDNF expression in these areas. The authors found that loss of BDNF in the DG or CA1 did not affect locomotor activity, contextual memory, cued memory, or baseline anxiety-like, depression-like and hedonic behaviors. However, deletion of BDNF in the DG, but not CA1, blocked the effect of ADs in a behavioral despair model. By contrast, local lentiviral knock down of BDNF in the rat dorsal DG results in anhedonic and depression-like behaviors (Taliaz et al., 2010). Signs of anhedonia were also seen when BDNF knock down was specifically targeted to the ventral subiculum, a region that has close connections with brain centers that regulate emotional behavior. However, when BDNF knock down was directed to the dorsal CA3 region of the HC, no effects were observed (Taliaz et al., 2010).

Chen et al. (2006) produced Val66Met SNP knock-in mice. Similar to human Met carriers, both hetero- (Val/Met) and homozygous (Met/Met) Met-mice showed decreased total hippocampal volume and impaired contextual memory (Chen et al., 2006). However, the cued memory and novel environment activity were similar in Met-mice and wildtype littermates. Furthermore, Met/Met mice showed increases in weight, aggressiveness, and anxiety-like behaviors. In addition, the effects of ADs in anxiety-related tasks were lost in these mice.

The Met-mice mice have also been tested in several other studies. Both Val/Met and Met/Met knock-in mice show impairments in a spontaneous olfactory discrimination test (Bath et al., 2008), while Met/Met mice show anxiety-like behavior that can be rescued by exposure to music (Li et al., 2010). In addition, Val/Met mice show vulnerability to stress induced anxiety- and depression-like behaviors, which can be rescued by some, but not all, ADs (Yu et al., 2012). Both human and murine Met allele carriers show impaired extinction of conditioned fear responses (Soliman et al., 2010). However, similar to other transgenic mouse models of BDNF, these mice have not conclusively clarified the connection between BDNF and the pathophysiology of depression.

Taken together, the large number of studies using mice with decreased BDNF expression has not yielded any clear connections between depression and BDNF. Several of the studies found opposing behavioral effects or no effects at all. Even though BDNF^(±)^ mice display a 50% reduction in BDNF levels throughout their entire lifespan, their behavioral phenotype is mild and varies from laboratory to laboratory. These discrepancies might be due to developmental compensatory mechanisms, variations in genetic backgrounds (Jacobson and Cryan, 2007), sex differences (Dalla et al., 2010), or inter-laboratory practices (Wahlsten et al., 2003). Furthermore, conditional KOs and mice with the Val/Met mutation have similar problems as BDNF^(±)^ mice. The behavioral results vary widely depending on the laboratory and the spatial location and temporal timing of the transgenes.

### Inhibition of TrkB signaling

BDNF^(±)^ mice and other approaches to decrease BDNF signaling have not provided any conclusive evidence regarding the connection between BDNF and depression-like behavior. Therefore, there is growing interest in genetic models of TrkB signaling (Table 3). Germline TrkB KO mice have serious developmental problems and die soon after birth (Klein et al., 1993; Rohrer et al., 1999). Only a few studies with heterozygous mice have been performed. Allen et al. (2005) found disrupted circadian rhythm in TrkB het mice. However, several conditional TrkB KO mouse lines have been produced and have permitted analyses of the behavioral effects of TrkB receptor deficiency that is spatially restricted to various brain regions.

**Table 3:** **Behavioral results of transgenic TrkB mice**.

**Mutant mice**	**Behavioral tests**	**Result**	**Background**	**Reference**
Forebrain-specific TrkB-receptor Knockout	EM, EZM, FST, NOR, OF	Increased locomotor activity in OF and mobility in FST. No changes in anxiety-like behavior	C57Bl/6N × 129/sv × CBA/J	Zörner et al., 2003
TrkB(±)	Spontaneus olfactory discrimination	Impairment in olfactory system	C57Bl/6	Bath et al., 2008
TrkB(±)	Circadian rythm	Disrupted circadian rythm	C57Bl/6	Allen et al., 2005
TrkB-KO in adult born neurons	EPM, OF	Decreased locomotor activity, increased anxiety-like behavior	C57Bl/6	Bergami et al., 2008
TrkB-KO in adult born neurons	NSF, TST	No behavioral changes, effects of ADs blocked	C57Bl/6	Li et al., 2008
Overexpressing catalytic TrkB receptor (TrkB.TK+), used as heterozygous	FST	Antidepressant-like behavior	CD2F1 (BALB/c × DBA/2)	Koponen et al., 2005
Overexpressing catalytic TrkB receptor (TrkB.TK+), used as heterozygous	CTA, EPM, FC, HP, LD, OF, RR, WM, Y-maze	Anxiolytic-like behavior, enhanced contextual and associative learning and memory. No changes in locomotor activity or coordination	CD2F1 (BALB/c × DBA/2)	Koponen et al., 2004
Overexpressing catalytic TrkB receptor (TrkB.TK+), used as heterozygous	OF, WM	Normal contextual learning and memory. Increased locomotor activity in male, but not in female mice	C57Bl/6	Kemppainen et al., 2012
Overexpressing truncated TrkB receptor (TrkB.T1, dominant negative), used as heterozygous	FST	No behavioral changes, effects of ADs blocked in heterozygous mice	CD2F1 (BALB/c × DBA/2)	Saarelainen et al., 2003
Overexpressing truncated TrkB receptor (TrkB.T1, dominant negative), used as heterozygous	WM	Impairment in contextual learning and memory	CD2F1 (BALB/c × DBA/2)	Saarelainen et al., 2000
Overexpressing truncated TrkB receptor (TrkB.T1, dominant negative), used as heterozygous	OF, WM	Normal contextual learning and memory	C57Bl/6	Kemppainen et al., 2012
Overexpressing truncated TrkB receptor (TrkB.T1, dominant negative), used as heterozygous	BW, SA	Normal social behavior; stress induces social avoidance. Normal body weight	C57Bl/6	Razzoli et al., 2011
TrkBF616A, time controlled inhibition of TrkB receptor	FC	Inhibition of TrkB receptor prior to testing prevents formation of fear conditioning	C57Bl/6	Johnson et al., 2008
TrkBF616A, time controlled inhibition of TrkB receptor		Inhibition of TrkB receptor prevents status epilepticus	C57Bl/6	Liu et al., 2013

Abbreviations: BW, body weight; CTA, conditioned taste aversion; EM, emergency test; EPM, elevated plus-maze; EZM, elevated zero maze; FC, fear conditioning; FST, forced swimming test; HP, hotplate; LD, light-dark box; NOR, novel object recognition; NSF, novelty suppressed feeding; OF, open field; RR, rotarod; SA, social avoidance; WM, water maze.

Crossing floxed TrkB KO mice with CamK-Cre mice produces mice with TrkB deficiency in the HC and forebrain neocortex starting at P15. These mice are viable and have normal brain morphology (Minichiello et al., 1999). They show a hyperactive phenotype with impulsivity but do not show baseline depression-like behaviors in models of behavioral despair (Zörner et al., 2003). They also do not display anxiety-like behavior. However, the antidepressant effects of ketamine were blunted in these mice (CamK-Cre; KO on P21) (Autry et al., 2011). Mice with specific TrkB deletion in adult DG neuronal progenitors (using nestin-CreERT2 mice) show decreased locomotor activity and increased anxiety-like behavior (Bergami et al., 2008). In addition, these mice do not respond to chronic AD treatment (Li et al., 2008).

As an alternative to decreasing TrkB signaling in mice through conditional deletion of TrkB receptors, Saarelainen et al. (2000) produced transgenic mice overexpressing the truncated, dominant negative form of the TrkB receptor (TrkB.T1; used as a heterozygous). When the TrkB.T1 receptor is under the control of the Thy1 promoter, it is overexpressed in postnatal neurons starting during the first few postnatal weeks (Saarelainen et al., 2000). When maintained on a CD2 (BALB/c × DBA/2) background, these mice display impaired long-term spatial memory (Saarelainen et al., 2000) and do not respond to the ADs imipramine and fluoxetine in behavioral despair models (Saarelainen et al., 2003). When these mice are backcrossed to a C57BL/6J background for more than 10 generations, they showed normal contextual learning and memory and normal social behavior (Razzoli et al., 2011; Kemppainen et al., 2012). However, these mice tend to avoid social situations when stressed (Razzoli et al., 2011). Interestingly, recent findings indicate that these mice show indifference toward the surrounding environment rather than depression-like behavior (unpublished data, Lindholm et al.).

Chen et al. (2005) used another approach to inhibit TrkB signaling and produced a knock-in mouse line with a single amino acid mutation near the ATP binding site of the TrkB receptor (TrkB^F616A^). This mutation does not influence the activity of the receptor, but if the pharmacologically inert kinase inhibitor 1NMPP1 is given (Bishop et al., 1999), then the activity of the TrkB receptor is inhibited. The advantage of these mice is that TrkB activity can be specifically, rapidly, and reversibly blocked without directly influencing the expression of the TrkB receptor. TrkB^F616A^ mice have been successfully used in Pavlovian conditioning model and epilepsy studies (Johnson et al., 2008; Liu et al., 2013), but large-scale behavioral studies using these mice have not yet been reported.

### Increased BDNF-TrkB signaling

Direct infusion of BDNF into the DG of rat HC leads to antidepressant-like effects (Shirayama et al., 2002). This finding indicates that increasing BDNF expression in the brain could also produce an antidepressant-like behavior. One study with mice overexpressing BDNF in excitatory neurons of the forebrain (HC, cortex, and amygdala) found an antidepressant-like behavioral phenotype with increased anxiety-like behavior (Govindarajan et al., 2006). Furthermore, chronic stress did not alter the anxiety-like behavior of these mice. Another group found that the same BDNF overexpressing mice showed increased anxiety-like behavior in some but not all tests (Papaleo et al., 2011). In the same study these mice demonstrated normal motor functions, contextual and cued fear memory, social behavior, and no alterations in depression-like behaviors. However, BDNF overexpressing mice have impairments in working memory and the auditory system. The results of these two studies are in part contradictory. However, the two studies used mice of different sex, which may explain the discrepancies, because sex is known to influence behavior of genetically modified mice (Monteggia et al., 2007; Autry et al., 2009). Another line of BDNF overexpressing mice showed improved performance in learning and memory tasks (Nakajo et al., 2008).

Transgenic mice overexpressing the full-length TrkB receptor (TrkB.TK+; used as a heterozygous) under the Thy1 promoter have enhanced TrkB signaling (Koponen et al., 2004). These mice show reduced anxiety- and depression-like behavior as well as improvements in contextual and associative learning and memory tasks without changes in locomotor activity or coordination (Koponen et al., 2004, 2005). In the forced swim test these mice behaved as if they had been treated with ADs, and AD treatment failed to produce any further effects (Koponen et al., 2005). These TrkB.TK+ mice were originally bred and maintained on a CD2 (BALB/c × DBA/2) background but were later backcrossed to the C57BL/6J background for more than 10 generations. However, when tested in the C57Bl/6J background, the phenotype was much milder (Kemppainen et al., 2012). Male mice showed increased locomotor activity while female mice did not differ from controls.

## Discussion

The roles of neurotrophins in depression and in the mechanisms underlying antidepressant effects have been widely studied. BDNF deficient mice are the most used model for these studies. These models have demonstrated a key role for BDNF-TrkB signaling in the mechanisms underlying the actions of ADs (MacQueen et al., 2001; Saarelainen et al., 2003; Chourbaji et al., 2004; Ibarguen-Vargas et al., 2009; Lindholm et al., 2012). Specifically, direct infusion of BDNF into the brain produces antidepressant-like behavior and enhances the effects of ADs, while blockade of BDNF signaling prevents the effects of ADs. It is possible that at least some of the antidepressant-like effects of BDNF are mediated through BDNF-induced long-term changes in synaptic connectivity, which in turn promote recovery from depression (Castren, 2005). The effects of acute BDNF are well-established in many regions of the HC and cortex (Messaoudi et al., 1998; Panja and Bramham, 2014), and chronic antidepressant treatment and BDNF infusion induce upregulation of an overlapping set of immediate early genes (Wibrand et al., 2006; Alme et al., 2007).

By contrast, there is no consensus as to whether decreases in BDNF activity cause depression-like behavior in mice since the results vary between different studies. The main difference between these studies is likely the temporal and spatial resolution of BDNF deficiency. However, other factors, such as developmental compensatory mechanisms, genetic backgrounds (Jacobson and Cryan, 2007), sex differences (Dalla et al., 2010), and inter-laboratory practices (Wahlsten et al., 2003) may also contribute to the variable results between studies.

Another possible reason for the failure to demonstrate an association between BDNF and mood disorders is that in most mouse models BDNF or TrkB are manipulated in most or all brain areas. However, there is evidence that manipulations of BDNF or TrkB signaling in different brain areas result in differential effects (Eisch et al., 2003; Berton et al., 2006). Therefore, widespread deletions could result in opposing effects that cancel each other out. Future studies with better temporal and spatial control of BDNF and TrkB will help to clarify this issue. Finally, mice are obviously not ideal for modeling depression and anxiety disorders. Local injection of a lentivirus coding for BDNF siRNA suggests that reduction of BDNF levels in rat HC produces depression-like behavior (Taliaz et al., 2010). Therefore, it is possible that genetically altered rats might produce more consistent results than mice when attempting to assess the role of BDNF in mood disorders. However, the Val66Met polymorphism in humans has not provided any clear evidence that altered BDNF release plays a role in depression and anxiety in humans. Thus, there may not be any direct association between BDNF and mood disorders.

In summary, while the importance of BDNF and TrkB in the antidepressant response is clear, further experiments in more refined mouse and rat models and in humans are necessary to clarify the role of BDNF signaling in depression and anxiety disorders.

### Conflict of interest statement

The authors declare that the research was conducted in the absence of any commercial or financial relationships that could be construed as a potential conflict of interest.
